# Salutatory

**Published:** 1889-08

**Authors:** F. W. McRae

**Affiliations:** Atlanta


### Editorial.

### SALUTATORY.

In assuming the management of this journal, it is with feelings of sadness that we retrospect the past, and see vacant the places once so well filled by the lamented Gray and Ashworth, men who have contributed so much to the success of the JOURNAL.

It shall be our purpose to keep the JOURNAL fully up to the high standard which it should maintain; and it shall be our constant endeavor to keep it in the future, as it has been in the past, the leading medical journal of the South—to make each succeeding issue better than the preceding. In the management of the JOURNAL, we shall strive to keep from its pages all that is merely provincial, and have it mirror the best medical thought of the day, without bias or prejudice. Its columns will always be open to those who have anything to say of general interest to the profession. We will devote especial attention to items and clippings from our best exchanges, so as to make that department reflect concisely what is going on in the medical world, its best thoughts and most interesting conclusions, ever keeping in view the fact that clinical demonstrations are of far more value to the busy practitioner than fine spun theories. We have perfected arrangements by which we will get correct reports of the best lectures of the leading clinicians of this country. Our special correspondents will also contribute newsy letters to every issue of the JOURNAL.

We shall admit into our advertising department only first-class advertisements. Books sent us for review will receive prompt and carefully written reviews. No change will be made in the editorial staff of the JOURNAL. Dr. Virgil O. Hardon, whose graceful editorials have contributed so much to the pleasure of the readers of the JOURNAL, will continue to have active control of that department. All existing contracts with the JOURNAL will be faithfully carried out.

In conclusion, we most respectfully solicit a continuance of the liberal patronage and kind indulgence which was extended to our predecessors, and which we shall constantly strive to merit.

F. W. McRAE.

*Atlanta, July 29, 1889.*

